# The host genetics affects gut microbiome diversity in Chinese depressed patients

**DOI:** 10.3389/fgene.2022.976814

**Published:** 2023-01-09

**Authors:** Ke Han, Lei Ji, Chenliu Wang, Yang Shao, Changfeng Chen, Liangjie Liu, Mofan Feng, Fengping Yang, Xi Wu, Xingwang Li, Qinglian Xie, Lin He, Yi Shi, Guang He, Zaiquan Dong, Tao Yu

**Affiliations:** ^1^ Key Laboratory for the Genetics of Developmental and Neuropsychiatric Disorders, Bio-X Institutes, Shanghai Jiao Tong University, Shanghai, China; ^2^ Shanghai Key Laboratory of Psychotic Disorders, Brain Science and Technology Research Center, Shanghai Jiao Tong University, Shanghai, China; ^3^ Asbios (Tianjin) Biotechnology Co., Ltd., Tianjin, China; ^4^ School of Mental Health, Jining Medical University, Jining, China; ^5^ Out-patient Department of West China Hospital, Sichuan University, Chengdu, China; ^6^ Mental Health Center of West China Hospital, Sichuan University, Chengdu, China; ^7^ Shanghai Center for Women and Children’s Health, Shanghai, China

**Keywords:** gut microbiome, depression, *SERPINA5*, beta diversity, microRNA

## Abstract

The gut microbiome and host genetics are both associated with major depressive disorder (MDD); however, the molecular mechanisms among the associations are poorly understood, especially in the Asian, Chinese group. Our study applied linear discriminant analysis (LDA) effect size (LEfSe) and genome-wide association analysis in the cohort with both gut sequencing data and genomics data. We reported the different gut microbiota characteristics between MDD and control groups in the Chinese group and further constructed the association between host genetics and the gut microbiome. *Actinobacteria* and *Pseudomonades* were found more in the MDD group. We found significant differences in the ACE and Chao indexes of alpha diversity while no discrepancy in beta diversity. We found three associations between host genetics with microbiome features: beta diversity and rs6108 (*p* = 8.65 × 10^–9^), *Actinobacteria* and rs77379751 (*p* = 8.56 × 10^–9^), and PWY-5913 and rs1775633082 (*p* = 4.54 × 10^–8^). A species of the *Romboutsia* genus was co-associated with the species of *Ruminococcus gnavus* in an internetwork through four genes: *METTL8*, *ITGB2*, *OTULIN*, and *PROSER3*, with a strict threshold (*p* < 5 × 10^–4^). Furthermore, our findings suggested that the gut microbiome diversity might affect microRNA expression in the brain and influenced *SERPINA5* and other spatially close genes afterward. These findings suggest new linkages between depression and gut microbiome in Asian, Chinese people, which might be mediated by genes and microRNA regulation in space distance.

## Introduction

Depression is one of the leading causes of disability, affecting more than 300 million people of all ages globally (Koopman et al., 2017), and it is a common, complex, and debilitating condition with a lifetime prevalence of 20% worldwide (Cai et al., 2020a). However, it is still a challenge to understand the mechanism of depression in pathology (Malhi and Mann, 2018).

The heritability of major depressive disorder (MDD) has been shown by twin and adoption studies (Flint and Kendler, 2014). The risk of depression varied among people with different genetic backgrounds. A meta-analysis identified 44 independent and significant loci, based on genome-wide association studies (GWAS) with 135,458 cases and 344,901 controls (Wray et al., 2018). The importance of genetic heterogeneity in depression has been verified by studies (Lee S. H. et al., 2013; Wray et al., 2018; Cai et al., 2020a; Cai et al., 2020b). Although some results could not coincide exactly, replicable findings of current GWAS helped inform the pathophysiology of major depressive disorder (Howard et al., 2018; Wray et al., 2018; Howard et al., 2021).

A growing body of research has indicated the bidirectional communication between the gut microbiota and the host’s central nervous system (CNS), also known as the microbiome–gut–brain (MGB) axis, which might help elucidate the pathophysiology of depression (Evrensel and Tarhan, 2021; Simpson et al., 2021). The alpha diversity and beta diversity showed differences in patients with the major depressive disorder from control groups (Liu et al., 2016; Chen Z. et al., 2018; Huang et al., 2018; Chung et al., 2019). *Actinobacteria* and *Bacteroidetes* at the phylum level commonly showed significant differences between the depressive disorders and controls (Rong et al., 2019; Dong et al., 2021; Simpson et al., 2021). Although the exact mechanism of the MGB axis has not been clarified, some processes associated with the immune system, metabolites, the endocrine system, and neuronal systems have already been reported (Dinan and Cryan, 2015; Rogers et al., 2016; Cenit et al., 2017; Waclawikova and El Aidy, 2018; Martins-Silva et al., 2021).

The gut microbiota composition could be modulated by intrinsic host factors, and the microbial GWAS have demonstrated different microbiome–host genotype associations (Weissbrod et al., 2018; Dubois et al., 2019). The association between *Actinobacteria* and rs4988235 (a locus in the *LCT-MCM6-ZRANB3* region), a typical example, was verified by high-level evidence, and meanwhile some other associations were reported in the same article (Qin et al., 2022). Although the association was supported by many sources of evidence, the mechanisms underlying the association are not fully understood.

microRNA (miRNA) was regarded as a mediator between the gut microbiome and intrinsic host factors. The expression of miR-294-5p, targeting some key genes in the kynurenine pathway, showed a difference between germ-free (GF) male mice and conventional mice, which was further normalized following colonization (Moloney et al., 2017). In the amygdala, the expression of miR-183-5p and miR-182-5p decreased in GF mice and was subsequently restored upon recolonization (Stilling et al., 2018). Furthermore, the miRNA expression in the brain and the gut microbiome is associated with depression or anxiety (Rosa et al., 2022). Antidepressant treatment for 8 weeks leads to increased plasma levels of miR-144-5p in patients with depression or anxiety compared to their pre-treatment baseline levels (Wang et al., 2015). Studies have researched the roles of miRNAs and microbiome in psychiatric disorders such as anxiety and depression (Meydan et al., 2016; Allen and Dwivedi, 2020; Huang and Wu, 2021).

This study aimed to construct the association between host genetics and the gut microbiome among MDD patients in the Asian group and find some hints of the specific mechanism underlying the association.

## Materials and methods

### Subjects

Patients with first-episode MDD and health controls (HCs) were recruited for this study. The participants were hospitalized patients recruited from the Mental Health Center, West China Hospital, Sichuan University, from January 2019 to October 2019. All samples were from Chengdu, Sichuan, China. We selected subjects with a normal body mass index (BMI, 18.5–22.9) to avoid bias, which might be caused by the influence of body weight on the intestinal flora.

The patients were diagnosed according to the Structured Clinical Interview for DSM-IV (SCID) by two psychiatrists (Aizawa et al., 2016). Patients aged less than 18 years or older than 45 years with organic etiology for their psychiatric symptoms, psychotic features, or intellectual disability were excluded.

According to the SCID, the HCs were 30 worker volunteers (aged 18–45 years), diagnosis of mental disorder was excluded by two psychiatrists, and their 24-item Hamilton Depression Rating Scale (HAMD-24) score was less than 7.

To further exclude the influence of physical diseases, subjects with the following diseases were excluded from our study: cardiovascular diseases (e.g., hypertension); digestive diseases (e.g., liver cirrhosis, irritable bowel syndrome, or inflammatory bowel disease); endocrine and metabolic diseases (e.g., diabetes mellitus, obesity, or fatty liver disease); drug or alcohol abuse; use of antibiotics, probiotics, prebiotics, or symbiotics during the 6 months before collection of fecal samples; known active bacterial, fungal, or viral infections; and obvious dietary preferences (e.g., vegetarians). A total of 51 eligible MDD patients and 30 HCs were recruited.

### Fecal sample collection and DNA extraction

Fecal samples were immediately frozen on collection in a sterile plastic cup and stored at −80°C before analysis. Microbial genomic DNA was extracted using the QIAamp DNA Stool Mini Kit according to the manufacturer’s instructions (Qiagen, Hilden, Germany). The 16S rRNA V3-V4 amplicons were generated using the National Institutes of Health (NIH) Human Microbiome Project protocols (16S 454 Sequencing Protocol HMP Consortium; https://www.hmpdacc.org). The V3–V4 region of the bacteria 16S ribosomal RNA genes was amplified PCR.

### 16S rRNA gene sequencing analysis

Libraries were prepared and subjected to paired-end sequencing with Illumina MiSeq by following Illumina’s standard protocol (Bartram et al., 2011). These QIIME2 16S rRNA sequencing protocols were used to pick and analyze operational taxonomic units (OTUs) (Kuczynski et al., 2012). Sequences from this project were deposited in the NCBI Short Read Archive under BioProject ID PRJNA647236.

### Bioinformatics and statistical analysis for gut microbiota

The sequence index file was used to identify and extract the sample data saved in the FASTQ format. Barcodes and the primers at the beginning and the end were used to identify and select sequence reads. The sequence number of each sample was normalized, and OTUs with 97% identity thresholds were used by the UPARSE (version 7.1 http://drive5.com/uparse/) software program. Chimeric sequences were identified and removed using UCHIME (version 4.1 http://drive5.com/uchime/). The taxonomy of each 16S rRNA gene sequence was analyzed by RDP Classifier (http://rdp.cme.msu.edu/) using the SILVA (SSU 138) 16S rRNA database at a confidence threshold of 70%. The further pathway abundances were predicted using PICRUSt2 (Douglas et al., 2020). Gut-microbiota-specific microbial characteristics (pathways or taxa) were analyzed using the linear discriminant analysis (LDA) effect size (LEfSe) method (http://huttenhower.sph.harvard.edu/galaxy/), which emphasizes both statistical significance and biological relevance.

Statistical analyses were performed using SPSS version 21(SPSS, Chicago, IL, United States). The t-test was used to compare the continuous variables following normal distribution. The Wilcoxon test was chosen for variables not following the normal distribution.

The α-diversity was calculated by the ACE, Chao, Simpson, and Shannon indexes. The β-diversity was calculated using the Bray–Curtis index as the distance method and reported according to principal co-ordinates analysis (PCoA) and non-metric multidimensional scaling (NMDS).

### Host SNP genotyping and GWAS quality control

Genomic DNA materials were extracted using QIAamp DNA Blood Mini Kit (51106, Qiagen, Hilden, Germany) from the peripheral blood of patients. WES was performed with the sequencing platforms (Genesky Biotechnologies, Shanghai, China, and WuXi NextCODE, Shanghai, China). The total WES depth was 100×, and the filtering procedure was performed in accordance with the manners reported previously (Luan et al., 2021). Sequencing data were annotated according to the GRCh37/hg19 reference genome. All MDD samples were genotyped with SNP arrays intending to provide exon-wide coverage of common variation. GWAS quality control and imputation were performed according to published PGC procedures (Wray et al., 2018). GWAS quality control was conducted separately for each sample. SNPs were removed for missingness ≥0.02, case–control difference in SNP missingness ≥0.02, gender difference (male ≥0.9, female ≥0.2), minor allele frequency ≤0.05, linkage disequilibrium (r^2^) <0.5, deprivation Hardy–Weinberg equilibrium test in controls <1 × 10^–6^, PIHAT values > 0.2, and heterozygosity deviation of three standard deviations.

### Genome-wide association analysis of gut microbiome features

We calculated the genetic principal components of ancestry from genome-wide genetic variants to estimate the population structure. PLINK 1.9 was used to perform a logistic regression model for taxa present in fewer than 90% of participants, adjusted for age, sex, and the first five genetic principal components of ancestry. For the taxa present in no fewer than 90% of participants, different pathways, and alpha diversity, we retained the quantitative value for analysis. The association assessment was fitted with the five genetic principal components of ancestry, age, and sex. Alpha diversity was calculated after randomly sampling 10,000 reads per sample.

The analysis for the genome-wide host genetic variants with beta diversity was performed using MicrobiomeGWAS (Hua et al., 2022), adjusted for covariates including the first five genetic principal components of ancestry, age, and sex.

The GWAS results were used to construct the network between genes and microbes or pathways. The lines in the constructed network represented the significant association between genes and microbes based on the *p*-values of the association test.

### microRNA–gene interactions

The data on miRNAs and target genes were obtained from miRTarBase (Huang et al., 2022). The database has accumulated >2,200,449 verified miRNA–target interactions (MTIs) from 13,389 manually curated articles and CLIP-seq data. An optimized scoring system is adopted to enhance this update’s critical recognition of MTI-related articles and corresponding disease information.

### Chromatin 3D model for associated genes

A chromatin 3D model based on molecular dynamics was used to discover the distance among genes in the nucleus center (Shi et al., 2020). The Euclidean distance was calculated to assess the possible interaction (closer proximity means more likely to influence each other). The 3D coordinate conversion was based on the Hi-C dataset of human embryonic stem cells (hESCs) with a resolution of 500 kb (bin size). We defined a close distance with means (M) minus 2 standard deviations (SD).

### Ethical approval statement

All procedures that contributed to this study complied with the ethical standards of national and institutional committees on human experimentation and with the Declaration of Helsinki (1975), as revised in 2008. All procedures that involved human subjects and patients were approved by the Ethics Committee of West China Hospital (WCH) of Sichuan University (approval number: 2019-268). All participants signed an informed consent form after appropriate oral information and explanation were provided.

## Results

### Gut abundance differs between depression and healthy cohorts

**FIGURE 1:**
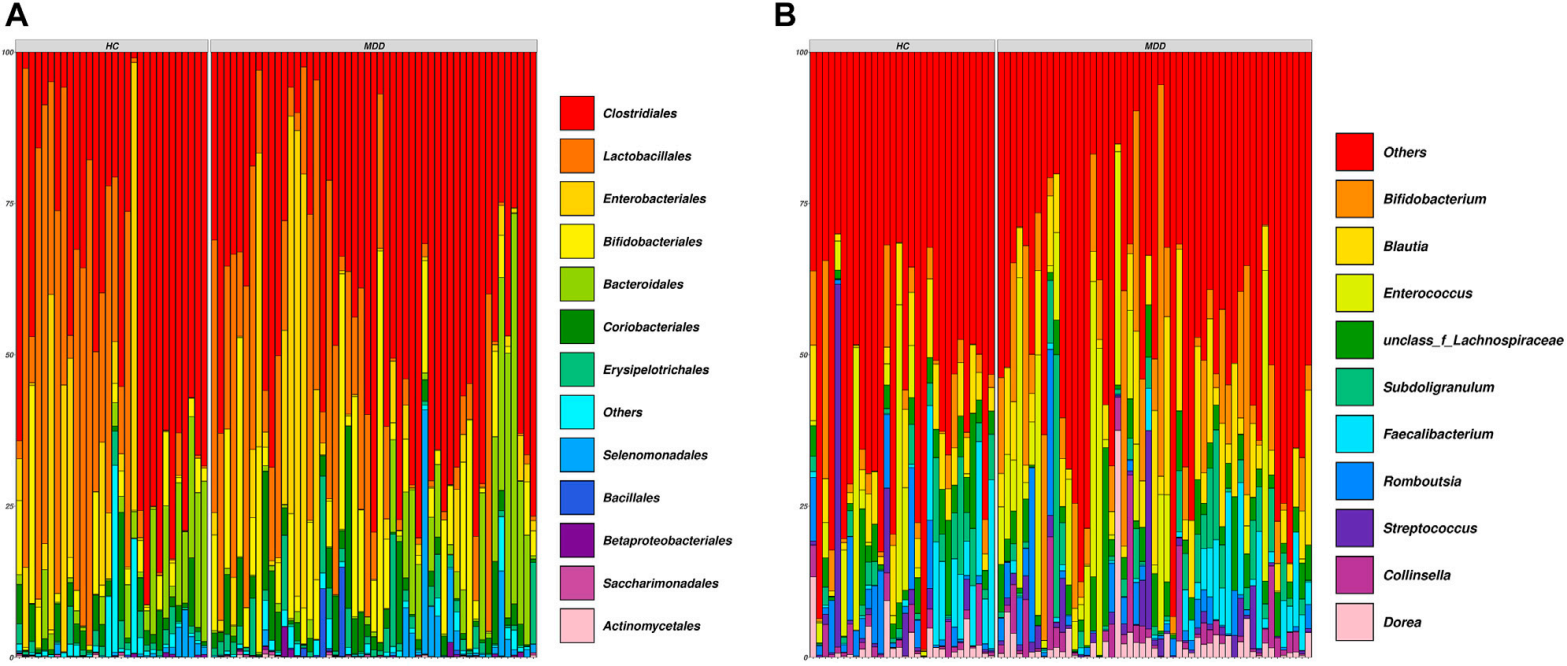
Composition of all samples in the cohort. **(A)** Family-level composition of samples. **(B)** Genus-level composition of samples. Each vertical line indicates one sample.

The demographic details are shown in Supplementary Table S1. Sequencing the V3–V4 regions of the 16S rRNA gene generated approximately 70.6 million sequences (median ± MAD): ∼51211 ± 6148 sequence reads per subject, and the denoised dataset contained 1249 unique sequences, covering 26 phyla, 55 classes, 118 orders, 214 families, 466 genera, and 671 identified species. The general overview of the abundance at the order and genus levels is shown in Figures 1A, B.

**FIGURE 2:**
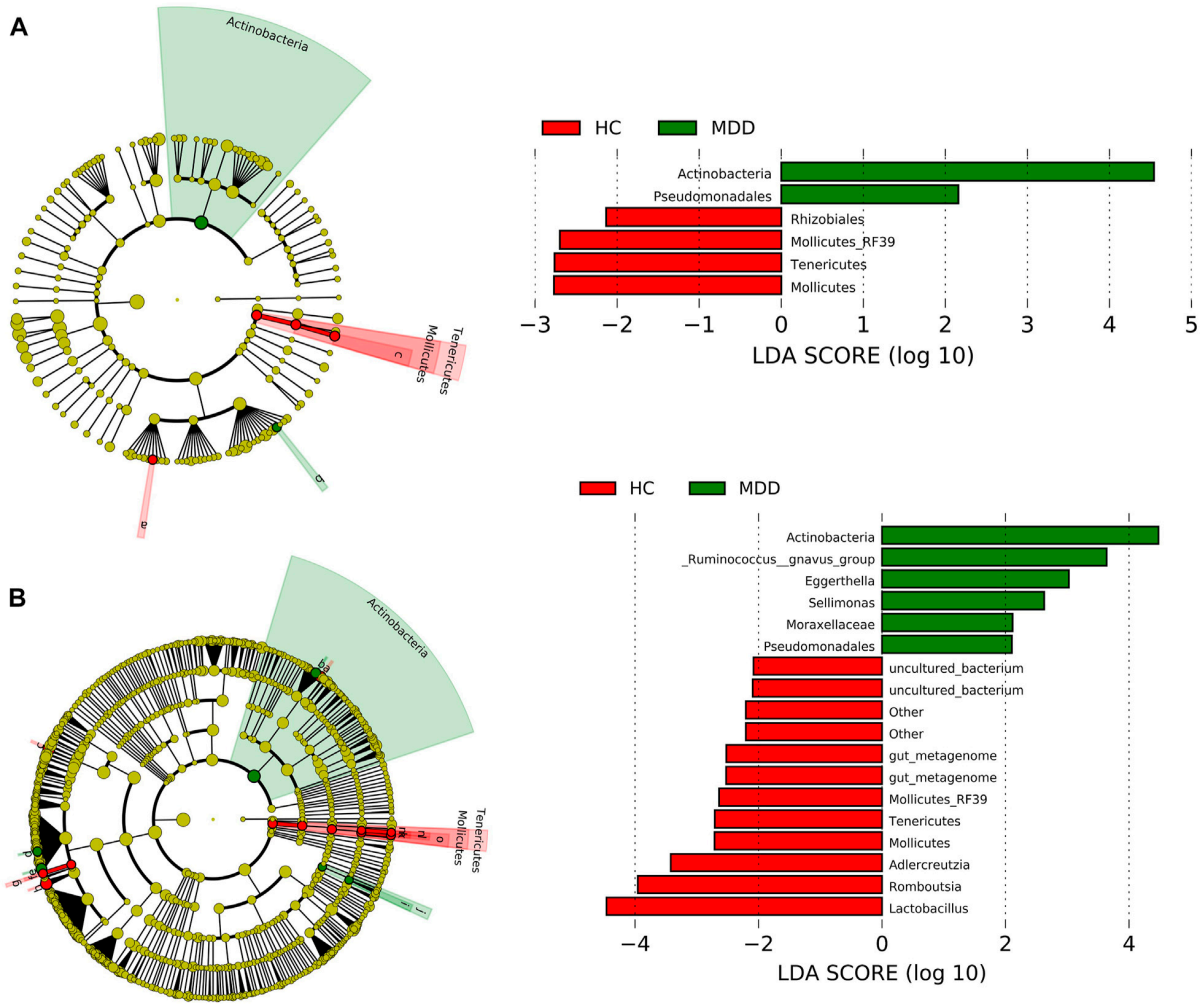
Linear discriminant analysis effect size (LEfSe) histograms and the cladograms for the comparisons of major depressive disorder (MDD) versus healthy controls (HCs). **(A)** LEfSe results at the order level. **(B)** LEfSe results at the genus level.

At the order level, *Clostridiales*, *Lactobacillales*, *Enterobacteriales*, *and Bifidobacteriales* were rich in both groups and showed no significance between groups. *Actinobacteria* and *Pseudomonadales* in the MDD group were higher than those in the HC group, while *Rhizobiales*, *Mollicutes_RF39*, *Tenericutes*, and *Mollicutes* were less in the MDD group (Figure 2A).

At the genus level, seven contents were higher in the MDD group, while 11 contents were higher in the control group (Figure 2B). *Actinobacteria* at the genus level showed the highest LDA score, 4.59 (*p* = 0.021), which indicated significant richness of *Actinobacteria* in the MDD group compared to the control group. *Lactobacillus* got the lowest LDA score, -4.42 (*p* = 0.037).

### The structure of the depressed microbiome in individuals with depression differs from the control subjects

**FIGURE 3:**
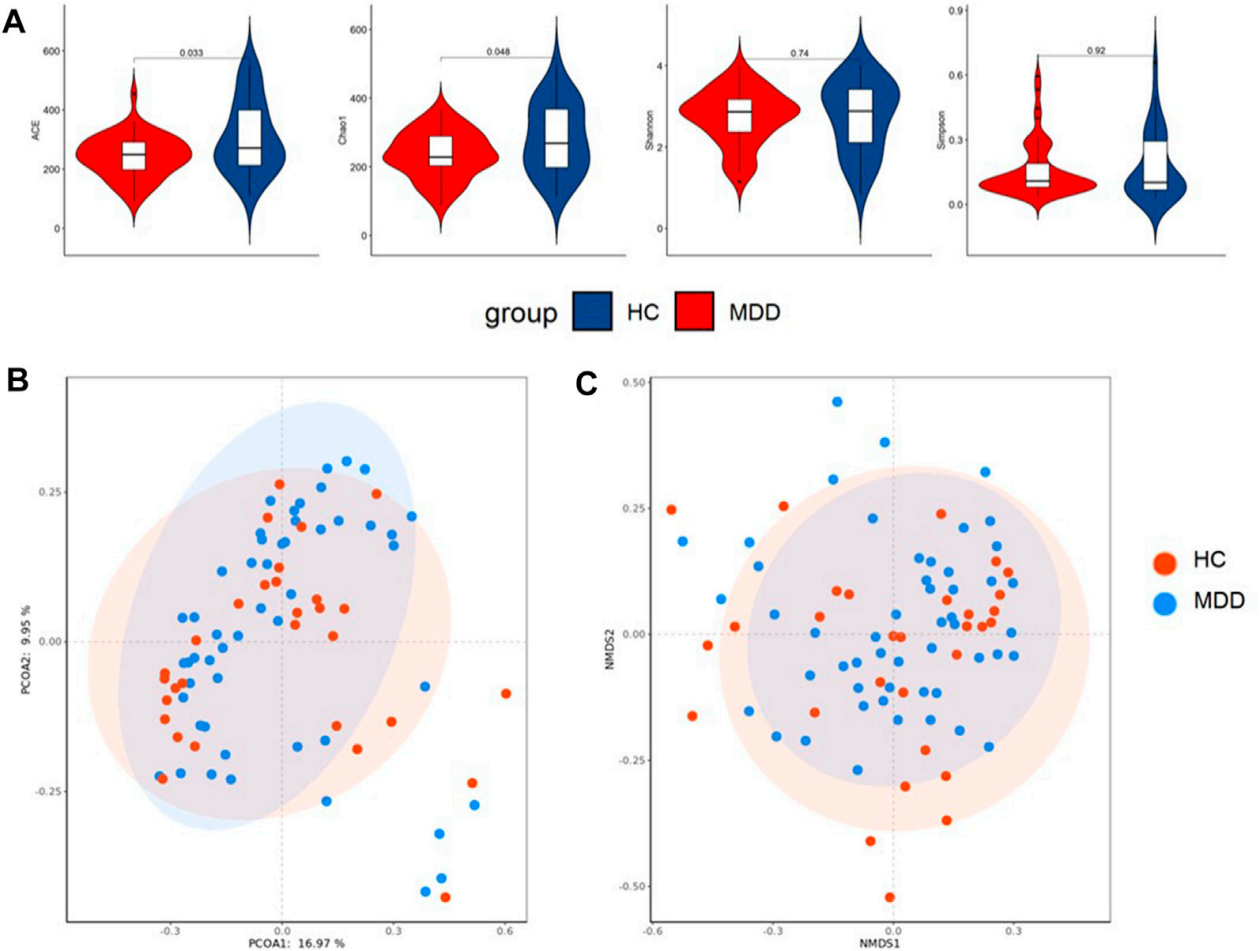
**(A)** Difference in alpha diversity between MDD and HC. **(B)** Result of principal co-ordinates analysis (PCoA) of beta diversity. **(C)** Result of non-metric multidimensional scaling (NMDS) of beta diversity.

Local diversity (α-diversity) could be used to investigate the structure of the gut microbiome. The community richness and diversity indexes, including Chao, ACE, Shannon, and Simpson indexes, were calculated to estimate α-diversity. The ACE and Chao indexes were significantly higher in healthy groups (*p* = 0.010; *p* = 0.026), while the Shannon and Simpson indexes showed no difference (Figure 3A).

PCoA and NMDS were used to test and visualize the difference in beta diversity between the control and case groups. Figure 3B indicates that PCoA showed no significant clustering between the cohorts (*p* = 0.143), and NMDS (Figure 3C) performed similar results.

### The pathways predicted differ between depression and healthy cohorts

**FIGURE 4:**
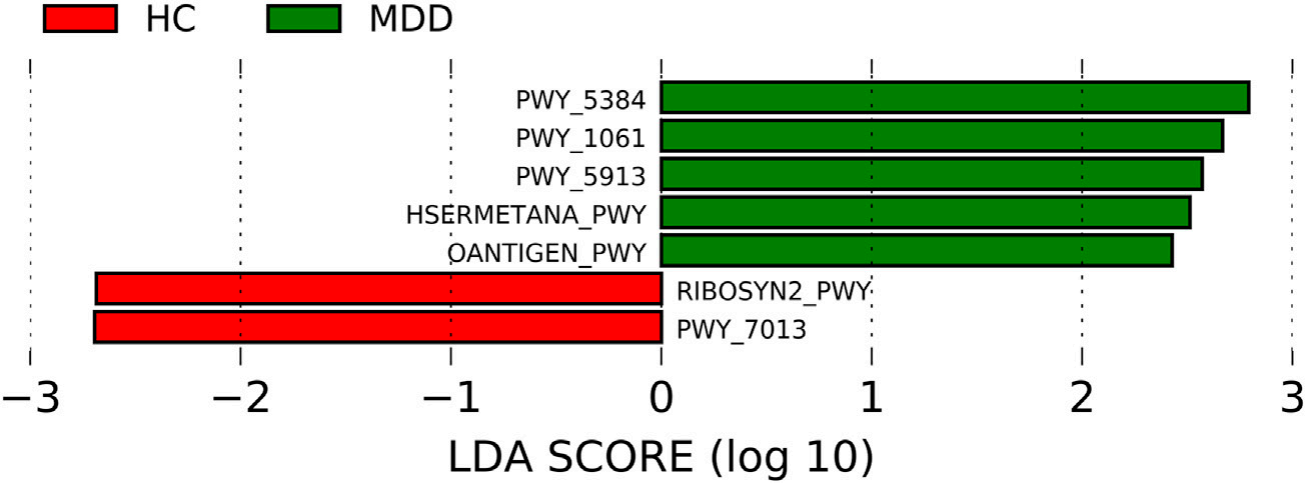
Linear discriminant analysis effect size (LEfSe) histograms for the comparisons of major depressive disorder (MDD) versus healthy controls of pathways.

The pathways were predicted using PICRUSt2. The differences in the pathways are shown in Figure 4. Five pathways were much more enriched in MDD: PWY-5384, PWY-1061, PWY-5913, HSERMETANA-PWY, and OANTIGEN-PWY. The most enriched pathway in MDD was PWY-5384 (the LDA score was 2.792, *p* = 0.002), which involved sucrose phosphorylase and indicated a discrepant use of energy. There were two pathways which were much more abundant: RIBOSYN2-PWY and PWY-7013, with LDA scores of -2.69 (*p* = 0.005) and -2.69 (*p* = 0.033), respectively. The correlation between significantly different pathways and microbiota is shown in Supplementary Figure S1, and 54 microbes at different levels were significantly correlated with the predicted pathways (74 in total).

### Association of host genetics with gut microbiome

**FIGURE 5:**
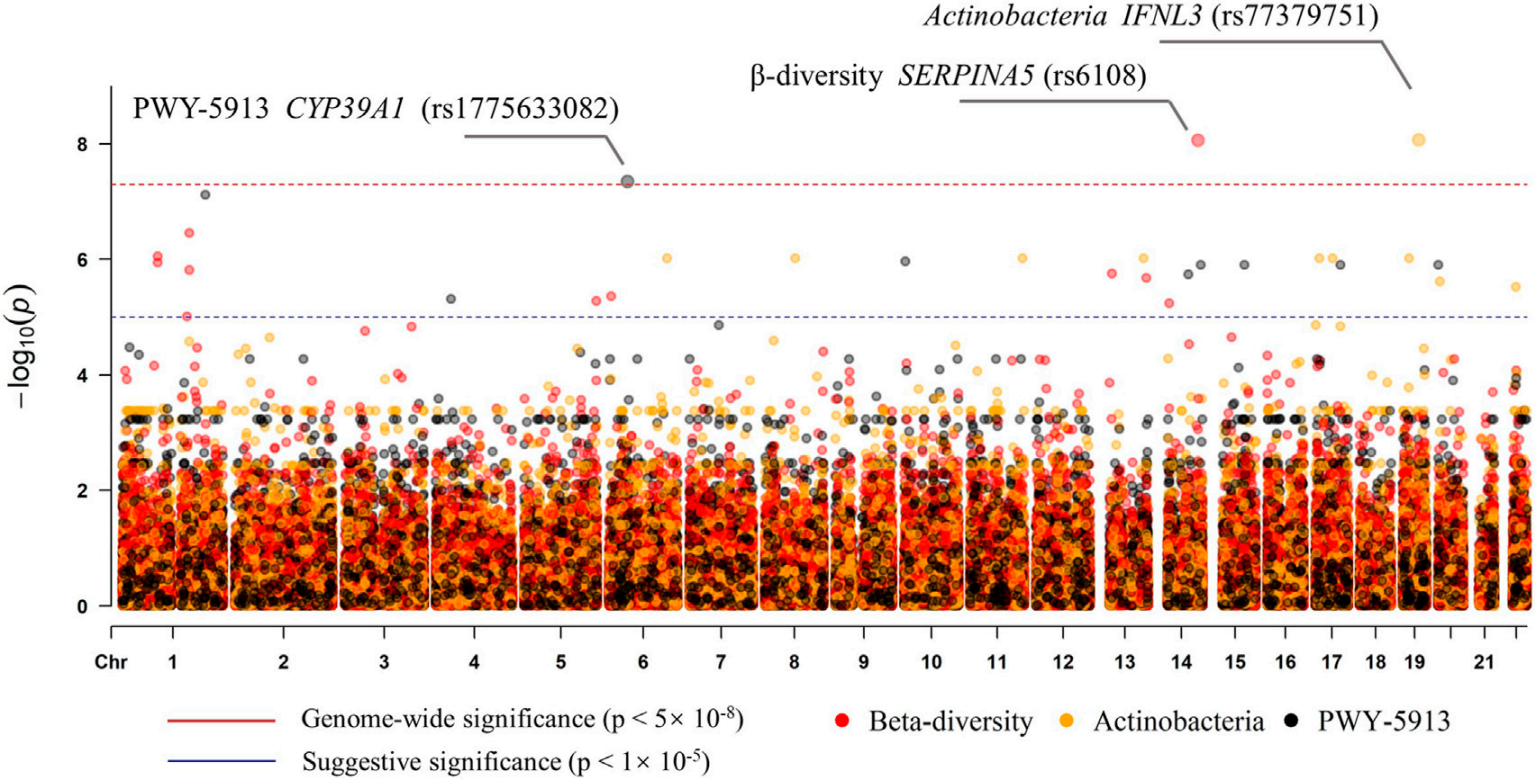
Manhattan plot of the associations with SNPs and the features (beta diversity, *Actinobacteria*, and PWY-5913).

To examine the association of host genetic variants with alpha diversity, we performed GWAS for four indexes, but no genome-wide significant signal (*p* < 5 × 10^–8^) was found. The beta diversity GWAS was performed using MicrobiomeGWAS based on Bray–Curtis dissimilarity. We found that one locus was associated with beta diversity at a genome-wide significant level (Figure 5): rs6108 was associated with beta diversity with a *p*-value of 8.65 × 10^–9^. The rs6108 is a three prime UTR variant, at *SERPINA5* gene. We subsequently performed discovery GWAS for individual gut microbes, which were significantly different. We found that only *Actinobacteria*, at the phylum level, was associated with host genetic variants (Figure 5) in one locus at the *IFNL3* gene (rs77379751; *p* = 8.56 × 10^–9^). The variant at rs77379751 is a missense variant with a deleterious function and predicts scores in both ROVEN (-6.88, cutoff = -2.5) and SIFT (0.02, cutoff = 0.05). Further association was explored between different pathways and genetics. We found that only PWY-5913 was associated with *CYP39A1* gene at the rs1775633082 locus (*p* = 4.536 × 10^–8^), passing the genome-wide test (Figure 5). The details of the genomic inflation factor for the GWAS data are shown in Supplementary Table S2.

### The internetwork of genes and microbes or pathways

**FIGURE 6:**
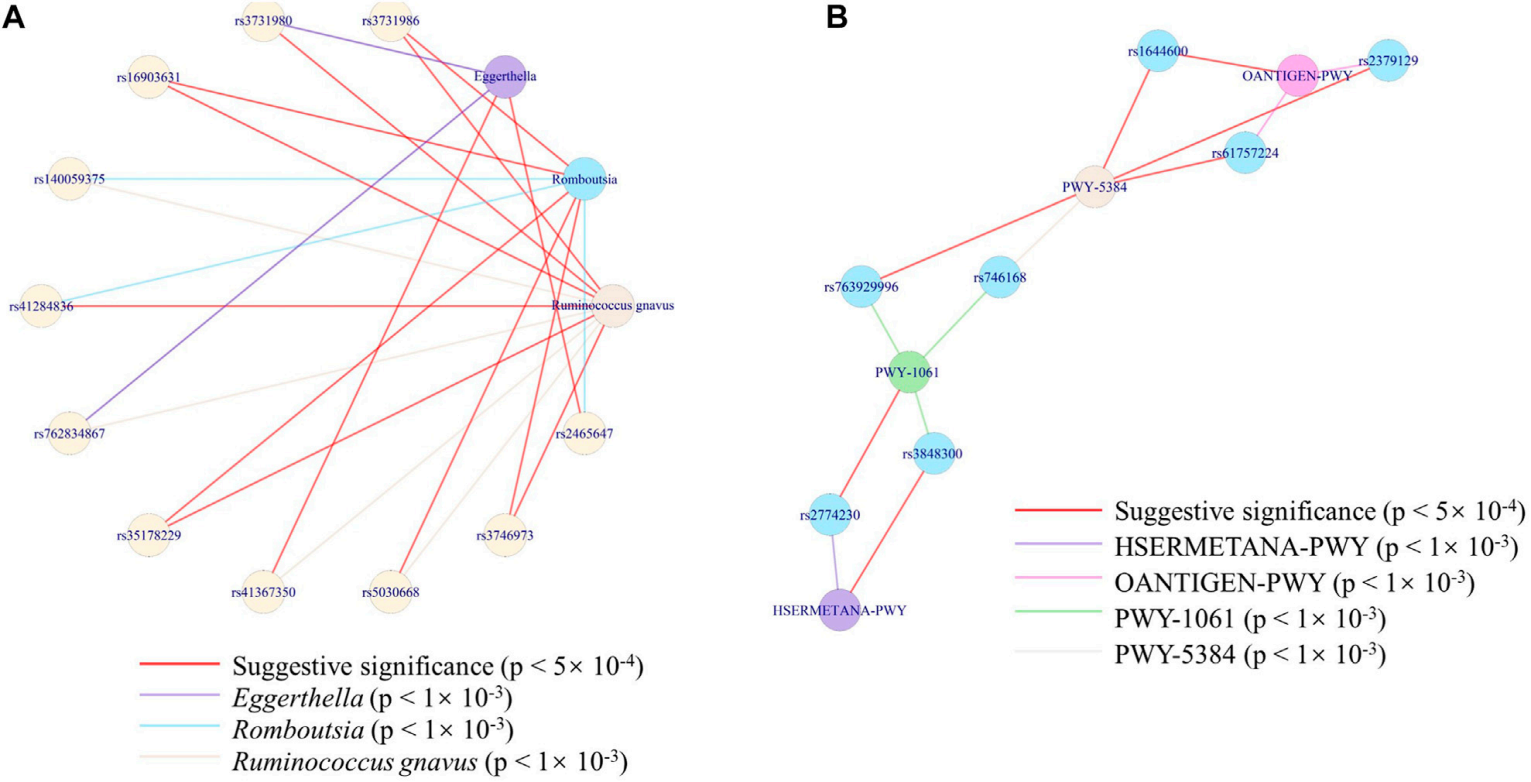
Network of the associations among SNPs and the features. **(A)** Network of the associations among three species with 12 genes at 13 loci. **(B)** Network of the associations among four pathways with six loci.

To further find the exact interaction between microbes/pathways and genes, we constructed the network between SNPs and microbes at the species level and the network between SNPs and pathways. We cannot find the connectivity at a rigorous level (*p* < 5 × 10^–8^). Considering the sample size, we took an effort at two suggestive significances (*p* < 5 × 10^–4^ and *p* < 1 × 10^–3^) to explore possible networks. A species (uncultured bacterium) of the *Romboutsia* genus was co-associated with another uncultured species of *Ruminococcus gnavus*, through *METTL8* (rs3731986), *ITGB2* (rs3746973), *OTULIN* (rs16903631), and *PROSER3* (rs35178229), at a strict threshold (*p* < 5 × 10^–4^). While with a loose threshold (*p* < 1 × 10^–3^), three species ( belonging to *Romboutsia* genus, *Ruminococcus gnavus*, and *Eggerthella* genus) were co-associated through 12 genes at 13 loci (Figure 6A). The connectivity between the species of *Ruminococcus gnavus* and *Eggerthella* genus was only through *USP28* (rs2465647). PWY-5384, PWY-1061, HSERMETANA-PWY, and OANTIGEN-PWY were co-associated through seven genes (Figure 6B), at the loose threshold (*p* < 1 × 10–3). Only PWY-5384 and OANTIGEN-PWY were co-associated with rs1644600 (a locus of *GSG1L* gene).

### MiRNA–gene network based on a close 3D distance

**FIGURE 7:**
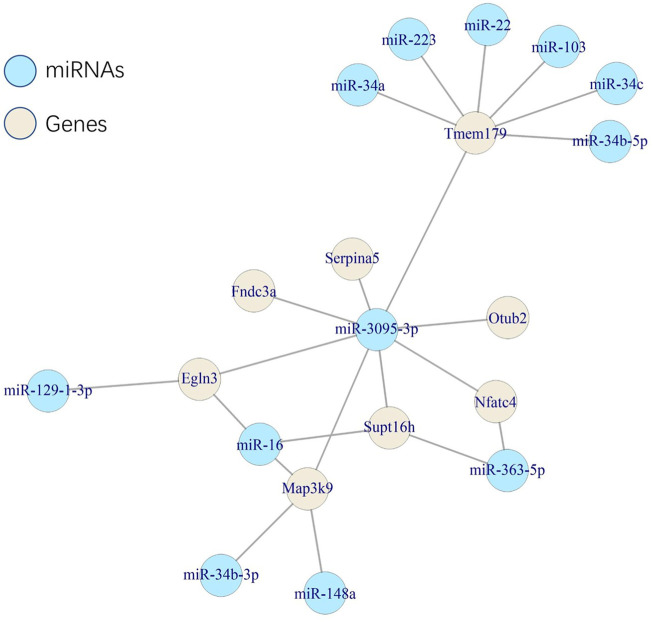
Network of the associations among microRNAs and genes.

We searched the microbe-associated genes (*IFNL3* and *SERPINA5*) to explore whether they interacted with miRNAs that were reported with depression/anxiety and microbiota. *SERPINA5* showed a target of mmu-miR-3095-3p, which was reported to be associated with anxiety-like behavior and microbiota (Rosa et al., 2022), while *IFNL3* was not. Following the interaction, we caught the targets of mmu-miR-3095-3p (737 targets in miRTarBase). Considering the possibility of interaction, we screened the targets based on a defined close distance (M-2SD) of the 3D model. Eight genes were finally left: *EGLN3*, *FNDC3A*, *MAP3K9*, *NFATC4*, *OTUB2*, *SERPINA5*, *SUPT16H*, and *TMEM179*. Also, all eight genes were found as at least one target of miRNAs which were reported to be associated with depression/anxiety and microbiota (Figure 7).

## Discussion

Our study showed the difference in microbiota between the patients with major depression disorder (MDD) and the controls. The phylum *Actinobacteria* was higher in MDD, which was consistent with prior results (Chen J. J. et al., 2018; Chen Z. et al., 2018; Chung et al., 2019; Lai et al., 2021). Some researchers reported that *Bacteroidetes* were higher in MDD (Jiang et al., 2015; Liu et al., 2016). At the order level, our results showed that *Pseudomonades* were higher in MDD, while other studies found results more on Enterobacteriaceae, despite the findings were not same (Jiang et al., 2015; Chen Z. et al., 2018). At the genus level, *Actinobacteria* was higher in MDD with the highest LDA score in our study, which was consistent with previous studies (Chung et al., 2019; Simpson et al., 2021).

As for alpha diversity, our results showed no difference in the Shannon index and Simpson index between MDD and control groups. Similarly, some studies found no difference between the MDD and control groups across all examined alpha diversity indexes (Chahwan et al., 2019; Mason et al., 2020), while some indicated that lower alpha diversity was found in depressive disorders using the Shannon index (Liu et al., 2016; Huang et al., 2018). The community richness was estimated by the Chao index and ACE index in our study, and both of them were significantly lower in the MDD group than in the control group, which was consistent with the previous results (Huang et al., 2018; Rong et al., 2019). Previous studies indicated that there would be significant differences in beta diversity between the MDD and controls, as shown by group clustering on PCoA (Chen Z. et al., 2018; Chung et al., 2019). However, we found no significant difference between the MDD and control groups using both PCoA and NMDS. Our findings were consistent with some other studies (Chahwan et al., 2019; Mason et al., 2020). On the whole taxa difference, our results were verified by previous results and gave some new findings. Based on the 16S rRNA data, predicted pathways also suggested some important insights. The differences in predicted pathways indicated the possible special mechanism of metabolism in the MDD group. The enriched pathways in MDD involved homogalacturonan biosynthesis, sucrose degradation, l-methionine biosynthesis, O-antigen building blocks biosynthesis, and the TCA cycle, which suggested a high level of glycometabolism and energy utilization. Some researchers have reported similar different factors of metabolic processes and emphasized the importance of the favorable metabolic status (Penninx and Lange, 2018; Wang et al., 2022). The significant correlations between predicted pathways and microbes were among 54 of 74 microbes at different levels, which showed that the key microbes were linked with key pathways. However, the correlation between PWY-5913 and microbes showed non-significance, which indicated that the functional-predicted pathways reflected an overall difference. The results suggested that microbiota could act not only through key individuals but also by affecting functional pathways as a whole.

The results of the association between microbiome characteristics and host genetic information indicated some specific links in the East Asian people. Our study used the threshold of *p* < 5 × 10^–8^ for the rigorous test, while the analysis discussed the nominal significance (*p* < 0.05) and suggestive significance (*p* < 5 × 10^–5^) to find results (Xu et al., 2020).

The association between *Actinobacteria* (at the phylum level) and rs77379751 (locus in the *IFNL3* gene) showed a deleterious prediction score in both ROVEN and SIFT. *IFNL3* gene encodes *IFN*-*λ3* protein, which is the one of the members of the type Ⅲ *IFN* family (Lasfar et al., 2016). It is reported that *IFN-λ* could play a crucial part in immune mechanisms that protect mucosal surfaces from pathogenic microbes by directly interacting more specifically with epithelial cells (Durbin et al., 2013; Mahlakoiv et al., 2015). The association between *IFN-λ* and depression is rarely reported. The *IFN* family, especially *IFN-α* and *IFN-γ*, is commonly associated with neuroinflammation in depression. Our results showed a possible link between *IFN-λ3* and depression, mediated by gut microbiome characteristics.

The association between beta diversity and the locus rs6108 indicated that genetic heterogeneity would influence gut microbiota in some way. Prior literature has reported some genetic variants that were associated with beta diversity, among which we found no significant association (*p* < 5 × 10^–8^), and these associations in our study could either survived Bonferroni correction (Bonder et al., 2016; Wang et al., 2016; Rothschild et al., 2018; Ruhlemann et al., 2018; Xu et al., 2020). For the various methods for sequencing and calculation of beta diversity, it is a challenge to verify and extrapolate the results across populations.

*SERPINA5* was indicated to be associated with beta diversity of gut microbiota. *SERPINA5* is a member of serpins. It was originally identified as an inhibitor of anticoagulant protease-activated protein C (aPC), and later it was shown with broad protease reactivity (Marlar and Griffin, 1980; Suzuki et al., 1983; Espana et al., 1989; Yang and Geiger, 2017). It is most mentioned that *SERPINA5* plays a role in hemostasis, male fertility, and cancer protection (Carroll et al., 1997; Elisen et al., 1998; Uhrin et al., 2000; Bijsmans et al., 2011; Lee E. K. et al., 2013; Jing et al., 2014). Only a few studies reported that *SERPINA5* had antimicrobial activity, which had been attributed to heparin-binding helix H (Malmstrom et al., 2009; Papareddy et al., 2014). However, few data *in vivo* supported the role of *SERPINA5* in the defense against bacteria (Yang and Geiger, 2017). *SERPINA5* was shown to be expressed in the hypothalamus (https://www.proteinatlas.org/ENSG00000188488-SERPINA5/brain), which might indicate a role in the hypothalamic–pituitary–adrenal (HPA) axis. It is reported that *SERPINA5* was associated with Alzheimer’s disease (AD) and Parkinson’s disease (PD) (Hurley et al., 2015; Crist et al., 2021). Although the linkage between AD and PD was indicated, we found no results which suggested that *SERPINA5* was associated with depression or gut microbiota.

Some studies reported that other proteins in the serpin family might be related to depression. *SERPINA6* was involved in HPA axis regulation and was associated with antenatal and postpartum depression (Skalkidou et al., 2019). *SERPINE1* and *SERPING1* were suggested crucial in the promotion of depression in patients with ovarian cancer (Yi et al., 2019). The association between the serpin family and the gut microbiome is not clear. A report hypothesized that *SERPINB5* (also known as maspin) might play a role in dysregulating the intestinal microbiota and inducing idiopathic inflammatory bowel disease (IBD)-related colorectal carcinoma (Gurzu and Jung, 2021). We all know about the linkage of inflammation with gut microbiota, but there was little evidence to show us the exact way that the serpin family works.

The network of microbiota and genes showed a complex regulation in the depression group. We found the co-association with four genes between the *Romboutsia* genus and *Ruminococcus gnavus* at the strict threshold (*p* < 5 × 10^–4^). It is reported that *ITGB2* had a powerful influence on immune cell infiltration into the acute myeloid leukemia (AML) tumor microenvironment (Wei et al., 2021). *OTULIN*, coding the M1-specific deubiquitinase, is essential for preventing TNF-associated systemic inflammation in humans and mice and critical for restraining life-threatening spontaneous inflammation and maintaining immune homeostasis (Damgaard et al., 2016; Hoste et al., 2021). The co-association between *IGTB2* and *OTULIN* of *Romboutsia* genus and *Ruminococcus gnavus* might influence depressive symptoms through a key role in immunization and inflammation. We found less evidence that *METTL8* and *DCAF17* were involved in the depression and complex microbiota interaction. The low significance of some connections suggested that the association could only play a weaker effect, and we should pay much attention to the cumulative effect.

As for the associations between predicted pathways and genes, we would like to emphasize the linkage between *CYP39A1* and PWY-5913. PWY-5913 was partial of the TCA cycle and commonly involved with energy utilization (Anderson et al., 2018; Linghu et al., 2022). Our results have shown no significant correlation between PWY-5913 and significantly different microbes, which indicated that this different pathway uncovered a whole-level difference. Meanwhile, *CYP39A1* encoded a member of the cytochrome P450 superfamily of enzymes and played an important role in tumor progression (Khenjanta et al., 2014; Ji et al., 2022) and neurodegenerative disorder (Vishweswaraiah et al., 2022). *CYP39A1* might affect a variety of microbes and finally show the difference in the PWY-5913 pathways. This association might indicate that a differentiated metabolism in MDD contributed to the depression symptom, which was consistent with the previous results (Zheng et al., 2016; Liu et al., 2021). It should be pointed out that these results were based on the prediction through 16S rRNA data, and the metabolism data needed further verification.

The specific connection of gut microbiota, genes, miRNAs, and depression or anxiety could be a new suggested way to improve symptoms. We found that *SERPINA5* was a target gene of mmu-miR-3095-3p, which is associated with the anxiety-like behavior (Chen et al., 2017). The authors found that compared to SPF mice, the mmu-miR-3095-3p in the hippocampus of germ-free mice was significantly downregulated, and the changes could be reversed following colonization. We emphasized the association between beta diversity of gut microbiota and *SERPINA5*, and the reported changes in mmu-miR-3095-3p were also related to gut colonization. Furthermore, the linkage between *SERPINA5* and mmu-miR-3095-3p was also reported. These findings construct a potential way that *SERPINA5* might be affected by mmu-miR-3095-3p, in turn influencing the gut microbiome, as a result of depression or anxiety-like behavior. The similar pathogenesis could not be found in *IFNL3*.

Furthermore, the miRNA–gene interaction showed a potential connection associated with space distance. Restricted by the space distance, we screened eight genes that could be the target of mmu-miR-3095-3p, and all the genes could be at least a target of miRNAs, which were reported to be associated with microbiota and depression/anxiety (Rosa et al., 2022). The results suggested a potential mechanism that miRNAs affected by microbiota in depression/anxiety showed a spatial specific effect, which indicated that specific space areas of chromatin might co-effect microbiome-associated depression/anxiety.

In conclusion, we reported the replicable results of the difference between the MDD patients and the control group and constructed the specific association of genes, miRNAs, microbiome, and depression/anxiety. Accumulated studies have demonstrated that gut microbiota and genes can influence depression by a bidirectional path, while our results give some original points. The specificity of microbiota in depression could be associated with fixed genes, and the genes might show spatial proximity, which indicated new points in the pathway for the potential treatment of depression. It is important that the key genes and microbiota among the pathways support a probability of personalized treatment of depression and could help researchers better understand the specific therapeutic targets.

We used 51 cases and 30 controls to find out the distinction between the gut microbiome and utilized only 51 cases to construct associations between the gut microbiome and genetic heterogeneity, which is considered a limitation to this study. It is also limited by the scale of cases and the results need larger research on the East Asian people for further verification. Due to the lack of data on diet and exercise, we were unable to further determine what roles they played in microbiota and depression. The deep exploration of how depression or anxiety was influenced by the network of genes and microbiota and the genes and miRNAs still need further studies.

## Data Availability

The original contributions presented in the study are publicly available. These data can be found at: PRJNA647236.
