# Supplementary material for: The host genetics affects gut microbiome diversity in Chinese depressed patients
Source: Front Genet. 2023 Jan 9;13:976814. doi: 10.3389/fgene.2022.976814 (PMC9868868; doi:10.3389/fgene.2022.976814)
Supplement: Supplementary file 1 [file Table1.DOCX]

**Extended Table 1.** Demographic description of the samples.

|  |  | MDD | HC | Statistic | P-value | Fisher p† |
| --- | --- | --- | --- | --- | --- | --- |
| Age |  | 27.14±8.98 | 29.23±6.57 | 5.85 | 0.16 |  |
| Weight |  | 57.71±12.07 | 57.55±7.38 | 6.31 | 0.94 |  |
| Height |  | 163.39±8.07 | 163.60±7.73 | 0.06 | 0.91 |  |
| BMI |  | 21.56±3.87 | 21.49±2.33 | 7.05 | 0.93 |  |
| Gender |  |  |  | 0.31 | 0.58 |  |
|  | Male | 14 | 10 |  |  |  |
|  | Female | 37 | 20 |  |  |  |
| Marriage |  |  |  | 0.14 | 0.93 | 0.92 |
|  | Never married | 29 | 16 |  |  |  |
|  | Married | 20 | 13 |  |  |  |
|  | Divorced | 2 | 1 |  |  |  |
| Inhabiting |  |  |  | 2.14 | 0.14 |  |
|  | Living alone | 14 | 13 |  |  |  |
|  | Cohabiting | 37 | 17 |  |  |  |
| Education |  |  |  | 3.53 | 0.32 | 0.38 |
|  | College degree or above | 39 | 22 |  |  |  |
|  | Secondary or senior high School | 8 | 4 |  |  |  |
|  | The junior middle school | 4 | 2 |  |  |  |
|  | Primary school and below | 0 | 2 |  |  |  |

Note: *^†^Fisher’s exact test is used if necessary.*

**Extended Table 2.** Inflation Factor of GWAS

| Feature | Inflation Factor |
| --- | --- |
| k__Bacteria_p__Actinobacteria | 0.905 |
| o__Clostridiales_f__Lachnospiraceae_g__.Ruminococcus..gnavus.group | 0.971 |
| o__Clostridiales_f__Peptostreptococcaceae_g__Romboutsia | 0.975 |
| o__Coriobacteriales_f__Eggerthellaceae_g__Eggerthella | 0.929 |
| f__Lachnospiraceae_g__.Ruminococcus..gnavus.group_s__ | 0.971 |
| f__Peptostreptococcaceae_g__Romboutsia_s__uncultured.bacterium | 0.975 |
| f__Eggerthellaceae_g__Eggerthella_s__uncultured.bacterium | 0.929 |
| k__Bacteria_p__Chloroflexi | 0.002 |
| k__Bacteria_p__Acidobacteria | 0.002 |
| k__Bacteria_p__Chloroflexi_c__Chloroflexia | 0.002 |
| k__Bacteria_p__Acidobacteria_c__Blastocatellia..Subgroup.4. | 0.002 |
| k__Bacteria_p__Tenericutes_c__Mollicutes | 1.276 |
| k__Bacteria_p__Tenericutes | 1.276 |
| k__Bacteria_p__Chloroflexi_c__Dehalococcoidia | 0.001 |
| p__Tenericutes_c__Mollicutes_o__Mollicutes.RF39 | 1.276 |
| p__Proteobacteria_c__Alphaproteobacteria_o__Rhizobiales | 1.020 |
| p__Proteobacteria_c__Gammaproteobacteria_o__Pseudomonadales | 0.986 |
| c__Mollicutes_o__Mollicutes.RF39_f__ | 1.359 |
| c__Mollicutes_o__Mollicutes.RF39_f__gut.metagenome | 1.449 |
| c__Gammaproteobacteria_o__Pseudomonadales_f__Moraxellaceae | 1.015 |
| o__Lactobacillales_f__Lactobacillaceae_g__Lactobacillus | 1.075 |
| o__Mollicutes.RF39_f__gut.metagenome_g__gut.metagenome | 1.449 |
| o__Coriobacteriales_f__Eggerthellaceae_g__Adlercreutzia | 1.001 |
| o__Mollicutes.RF39_f__uncultured.bacterium_g__uncultured.bacterium | 0.044 |
| o__Clostridiales_f__Lachnospiraceae_g__Sellimonas | 0.971 |
| o__Betaproteobacteriales_f__Burkholderiaceae_g__Ralstonia | 1.042 |
| f__Ruminococcaceae_g__.Eubacterium..coprostanoligenes.group_s__metagenome | 1.330 |
| c__Mollicutes_o__Mollicutes.RF39_s__ | 1.359 |
| f__Eggerthellaceae_g__Adlercreutzia_s__uncultured.bacterium | 1.001 |
| f__Ruminococcaceae_g__Ruminococcus.1_s__metagenome | 1.214 |
| f__Lactobacillaceae_g__Lactobacillus_s__ | 0.987 |
| f__Lachnospiraceae_g__Sellimonas_s__uncultured.bacterium | 0.971 |
| f__Lachnospiraceae_g__Lachnoclostridium_s__.Clostridium..scindens | 1.000 |
| PWY-5384 | 1.024 |
| PWY0-1061 | 0.968 |
| HSERMETANA-PWY | 1.051 |
| PWY-5913 | 1.001 |
| OANTIGEN-PWY | 1.012 |
| PWY-7013 | 0.631 |
| RIBOSYN2-PWY | 1.017 |
