# Supplementary figures and images for: The host genetics affects gut microbiome diversity in Chinese depressed patients
Source: Front Genet. 2023 Jan 9;13:976814. doi: 10.3389/fgene.2022.976814 (PMC9868868; doi:10.3389/fgene.2022.976814)

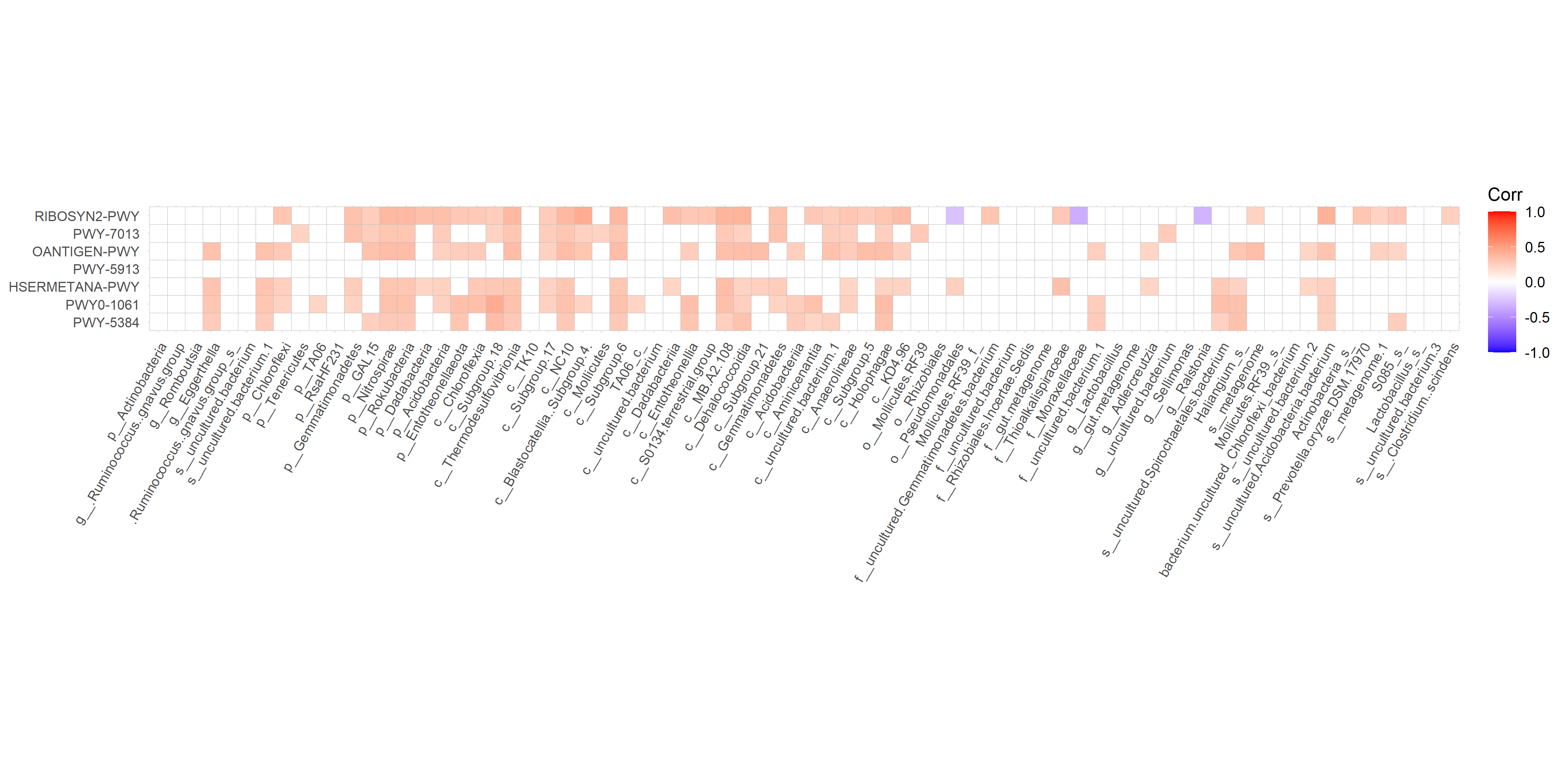

Supplement: Supplementary file 2 [file Image1.PNG]
